# Resistin Production from Adipose Tissue Is Decreased in db/db Obese Mice, and Is Reversed by Rosiglitazone

**DOI:** 10.1371/journal.pone.0065543

**Published:** 2013-06-12

**Authors:** Hongying Ye, Herbert J. Zhang, Aimin Xu, Ruby L. C. Hoo

**Affiliations:** 1 Department of Medicine, The University of Hong Kong, Hong Kong, China; 2 Research Center of Heart, Brain, Hormone, and Healthy Aging, The University of Hong Kong, Hong Kong, China; 3 Department of Endocrinology, Huashan Hospital, Fudan University, Shanghai, China; Central China Normal University, China

## Abstract

**Objective:**

This study was designed to (1) investigate the expression profiles of resistin in db/db mice and its dynamic association with metabolic parameters; and (2) evaluate the effects of Rosiglitazone on production of resistin.

**Methods:**

Db/db mice and their lean litter mates were used for this study. Epididymal fat tissue was excised from mice of different age (from 5 to 12 weeks) for ex vivo incubation. Resistin,along with adiponectin,in serum and conditioned culture medium of epididymal fat pads were measured with immunoassays. The gene expression of resistin was determined by real-time PCR. Rosiglitazone or the vehicle (PBS) was administered into db/db mice by daily intra-gastric gavage. Differentiated 3T3-L1 adipocytes were used for in vitro evaluation.

**Results:**

The secretion of resistin from the fat pads in db/db mice was significantly lower than that in lean mice (P<0.01). The mRNA expression of the resistin gene in fat tissue of db/db mice at the age of 5 weeks was decreased by 60.5% compared to lean controls (p<0.05). Serum levels of resistin were comparable between the obese and lean groups, perhaps due to the increased total fat mass in db/db mice. Correlation analysis showed that serum resistin levels were positively correlated to resistin secretion from fat pads(r = 0.844,P = 0.000), while negatively associated with the body weight (r = −0.515, P = 0.000) and fasting glucose level (r = −0.357, P = 0.002). Notably, treatment with rosiglitazone increased the serum resistin levels by 66.4%(P<0.05)in db/db mice. In 3T3-L1 adipocytes, Rosiglitazone (10 uM) markedly enhanced the secretion of resistin by 120% (P<0.01) and its gene expression by 78.1% (P<0.05).

**Conclusion:**

Both resistin gene expression and its secretion from the epididymal adipose tissue were decreased in db/db obese mice, while the insulin-sensitizing drug rosiglitazone increased resistin production. Our results do not support the role of resistin as an etiological link between obesity and diabetes.

## Introduction

Adipose tissue(AT) is known to produce a vast array of adipokines, which are implicated in the pathogenesis of the metabolic syndrome. Such factors include TNF-α (tumour necrosis factor-α), leptin, adiponectin, IL (interleukin) −6 and resistin [1]–[3].

Resistin, an adipocyte-derived signaling polypeptide, was originally identified in 2001 [4], [5]. Initial studies [5] showed that circulating resistin levels were increased in diet-induced and genetic obesity mice and decreased by the anti-diabetic drug rosiglitazone. Administration of anti-resistin antibody improved blood sugar and insulin action in mice with diet-induced obesity. Moreover, treatment of normal mice with recombinant resistin impairs glucose tolerance and insulin action. These observations characterized resistin as a potential etiological link between obesity and diabetes.

However, to date there has been considerable inconsistent reports from the animal and clinical studies for or against the role for resistin as an important pathogenic factor in obesity, insulin resistance and type 2 diabetes [6], [7]. There are many results that support the role of resistin as an important pathogenic factor in obesity and related disorders: higher serum level of resistin in the various murine genetic or diet-induced model of obesity [5]; positive correlation between the resistin serum level, gene expression level and BMI, degree of insulin resistance [8]; down-regulation by Rosiglitazone treatment [9], [10].

There were also many controversial results against the role of resistin in the relation between obesity and its related diseases. Way JM et al [11] firstly reported that resistin gene expression was significantly decreased in the white adipose tissue (WAT) of several different models of obesity including the ob/ob, db/db, tub/tub, and KKA(y) mice compared with their lean counterparts. Furthermore, in response to several different classes of antidiabetic peroxisome proliferator-activated receptor (PPAR)-γ agonists, resistin gene expression in WAT was increased in both ob/ob mice and Zucker diabetic fatty rats. Furthermore, an improvement of insulin sensitivity after PPAR-α agonist treatment is accompanied by paradoxical increase of circulating resistin Levels [12]. In human, Janowska J [13] and Pfutzner A [14] reported that serum resistin concentrations did not correlate with BMI, HOMA, fasting plasma glucose level, or fasting plasma insulin level, and Wasim H et al found that there were no significant difference among NGT, IGT and new diagnosed type 2 DM [15], plasma levels of resistin in subjects with CAD or diabetes are similar to the controls [16].

Therefore, the role of resistin in obesity-associated insulin resistance remains unclear.

In this study, we investigated the expression profiles of resistin of AT in db/db obese/diabetic mice at different age (from 5 to 12 weeks) and its association with metabolic parameters, and evaluated the effects of Rosiglitazone on production of resistin. And the time courses of adiponectin in serum and production from the fat pads were investigated at the same time.

## Materials and Methods

### Animals

Male C57BKS db/db diabetic mice and lean litter mates at the age of 5,6,7,8,10 and 12 weeks were used in this study(the numbers of db/db mice at each time point were 9,5,5,6,5 and 6; while the numbers of lean control were 8,6,8,7,5,and 5). The mice were housed in a room under controlled temperature (23±1°C) and a light-dark cycle (12∶12), with free access to water and standard mouse chow. Intraperitoneal glucose tolerance test (ipGTT) was performed to assess the glucose metabolic situation 3 days before sacrifice. Blood glucose was checked from the tail vein with glucosemeter (*ACCU-CHEK®*, Roche) before and 10, 20, 30,45, 60,75,90 min after the glucose injection intraperitoneally at a dose of 1 mg/g. At the sacrificing day, after a 6h fast, body weight was recorded; blood sample was collected through cardiac puncture and serum was separated and stored at −20°C for further assay. Epididymal, perirenal and abodomal AT were collected and weighted, epididymal AT was freshly prepared for explants incubation and frozen then stored for RNA analysis.

Rosiglitazone (20 mg/kg/day) or the vehicle (PBS) was administered into other 10 db/db mice at the age of 10 weeks by daily intra-gastric gavage for 4 weeks. Random blood glucose (RBG) was checked from the tail vein with glucosemeter (*ACCU-CHEK®*, Roche). Blood sample was collected through cardiac puncture and serum was separated and stored at −20°C for resistin assay.

All experiments were approved by the Animal Ethics Committee of the University of Hong Kong and conducted under the institutional guidelines for the humane treatment of laboratory animals.

### Adipose Tissue Explants Incubation [17]

In db/db mice, epididymal AT(200∼300 mg) was collected in duplicate while adipose tissue from 3∼5 lean mice were pooled together for incubation. The handling of tissue was done under aseptic conditions. After rinsed with prewarmed PBS and cut with scissors into small pieces (10–20 mg), explants of AT were incubated in Dulbecco’s modified Eagle’s medium/Ham’s F12 (1∶1, Sigma No. 2906) containing 25 mM Hepes, 2.4 mM sodium bicarbonate, 10 mg/ml bovine serum albumin, 10,000 U/ml Penicillin, 10 mg/ml Streptomycin, 25 ug/ml amphotericin B and 55 µM ascorbic acid(The pH of the buffer was adjusted to 7.2 and then filtered through a 0.2-µm filter) for 30 min in water bath orbitally shaking at 80rpm(Grant OSL200) at 37°C with air as the gas phase, then centrifuged for 30 s at 400 *g* to remove erythrocytes and pieces of tissues. The explants were separated from the medium plus the sedimented cells and resuspended in fresh buffer. The explants (200 mg/ml) were then incubated for 24h in suspension culture under aseptic conditions. After 24h incubation, medium was collected and stored at −20°C for further assay.

### Cell Culture

3T3-L1 cells were cultured in Dubecco’s modified Eagle’s medium (Gibco) supplemented with 10% feta bovine serum. Adipocyte differentiation was induced as we previously described [18].The adipocyte conditioned medium was collected after 48h incubation with or without Rosiglitazone (10 uM). Meanwhile, the adipocytes were collected in Trizol for further RNA evaluation.

### Immunoassay

Resistin level in serum, explants incubation medium(EIM) and conditioned medium from 3T3-L1 adipocytes was measured using an in house ELISA assay. Capture antibody for resistin (R&D system, MAB1069), biotinylated detection antibody (R&D system, BAF1069) and standard (R&D system, 1069-RN) were purchased from R&D systems, Inc. The assay was performed according to the manufacturers’ protocols. Adiponectin level in serum and EIM were measured using an in house ELISA assay [19].

### Evaluation of the Resistin Gene Expression Levels by Quantitative Real-time RT-PCR

Total RNA was extracted from adipose tissue and adipocytes using Trizol reagent (Invitrogen). One microgram of total RNA was transcribed into cDNA using ImProm-II Reverse. Each cDNA sample was analyzed for gene expression by quantitative real-time PCR using the fluorescent TaqMan 5′-uclease assay on an Applied Biosystems Prism 7000 sequence detection system. The TaqMan real-time PCR was performed using 20×TaqMan Master Mix and 20×assay-on-demand TaqMan primers and probes (Applied Biosystems). Analysis was performed with ABI Prism 7000 SDS Software. The sequences of primers are: Forward 5′-GGCTCTGTGCTCCTCCATCT-3′, Reverse 5 ’-AGAGTCGTTGACGTTATCTGCATAG-3′, Probe FAM-59-CCCATACACCTGGAGCCAGACTTGGT-39-TAMRA.

The homeostasis model assessment (HOMA) of insulin resistance (HOMA-IR) was calculated with the following formula: fasting plasma glucose (FPG, mmol/L) ×fasting insulin (FINS, mIU/L)/22.5, HOMA of beta-cell function (HOMA-B) with 20×FINS/(FPG-3.5).

### Statistics

All analyses were performed with the Statistical Package for Social Sciences version 14.0 (SPSS, Chicago. IL). Data are expressed as means± standard deviation (SD). The inter-group comparisons were made by Student’s t test or one-way ANOVA. In all statistical comparisons, a P value of less than 0.05 was considered statistically significant. The experiments in cell culture were reproduced in three independent experiments.

## Results

### 1. Time Course of Body Weight, Adipose Tissue, Glucose, and Circulating Resistin Level in db/db Mice

**Figure 1:**
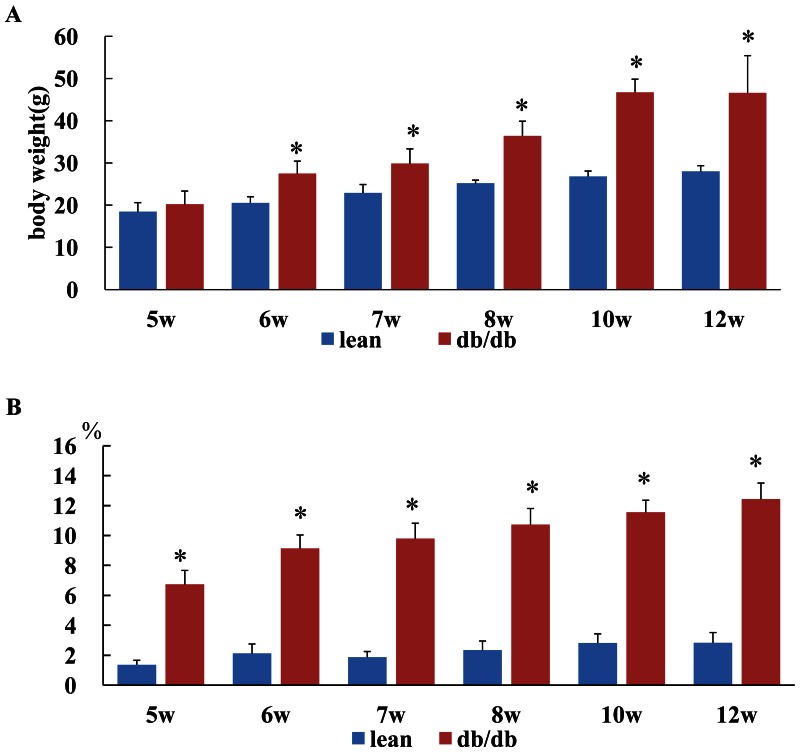
The time course of body weight (BW) and total adipose tissue (tAT) percentage of BW. At the age of 5 week, BW was comparable between db/db mice and lean control. Db/db mice older than 6W had higher BW than lean control (Figure 1A). At each time point, tAT(all AT from epididymal, perirenal and abodomal) percentage of body weight(BW) from db/db mice were significantly higher than lean control(Figure 1B), indicating that fat tissue mass increased markedly in db/db mice from 5 weeks old.*P<0.01,compared with lean control at the same age.

At the age of 5 weeks, body weight (BW) was comparable between db/db mice and lean control while adipose tissue weight of epididymal, perirenal and abdominal subcutaneous depots and its percentage of BW from db/db mice were significantly higher than lean control, indicating that fat tissue mass increased markedly in db/db mice. Along with the growing, db/db mice had higher BW and higher percentage of adipose tissue (Fig. 1A,1B).

**Figure 2:**
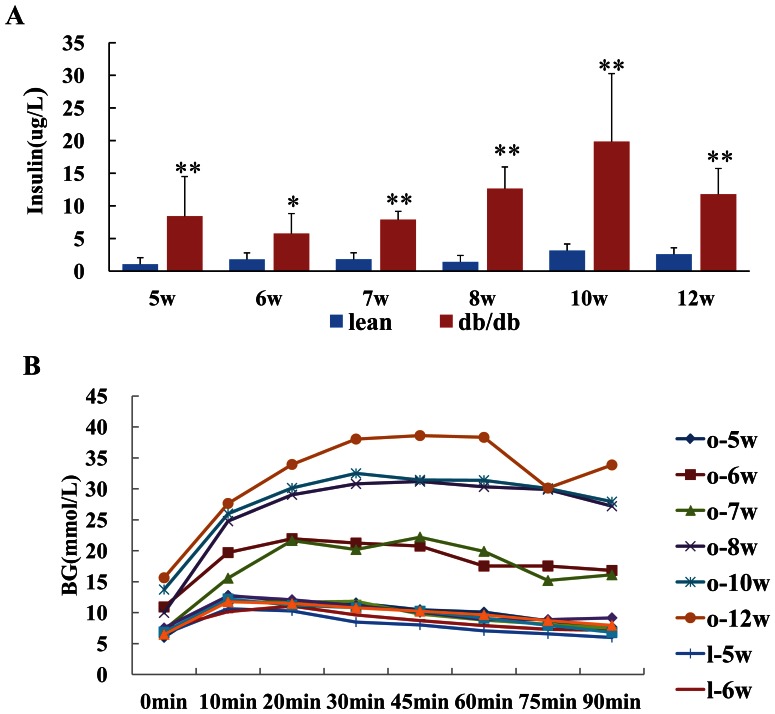
Time course of serum insulin level and glucose metabolism situation of mice. Figure 2A showed that db/db obese mice had higher level of insulin at each time point than lean control. * p<0.05 **p<0.01 *vs* lean control at same age. Compared with the lean group, db/db obese mice had normal response to intraperitoneal glucose tolerance test(ipGTT) at the age of 5w, had impaired ipGTT at the age of 6w and increased fasting glucose level at the age of 10w (Fig. 2B).

Compared with the lean group, db/db mice had normal response to ipGTT but increased insulin level at the age of 5 weeks, had impaired ipGTT (IGT) at the age of 6 weeks and increased fasting glucose level at the age of 10 weeks (Fig. 2A, 2B).

**Figure 3:**
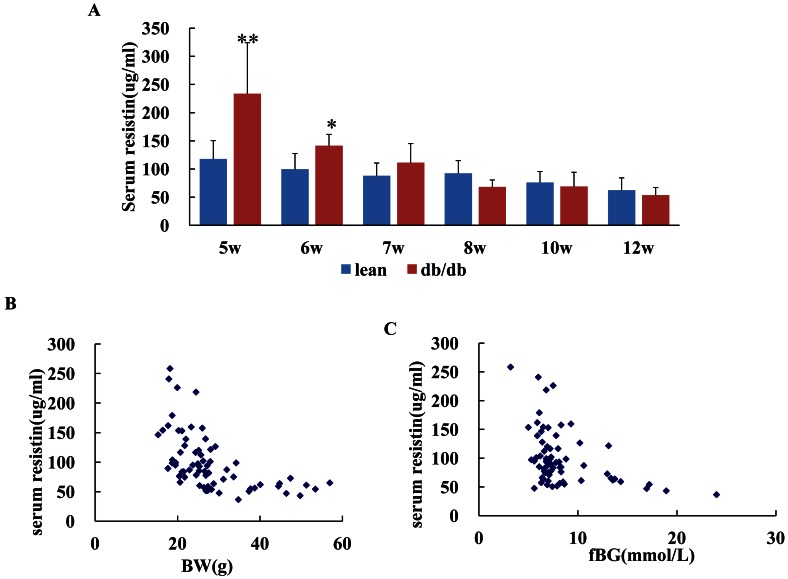
Time course of circulating resistin level at different age and its relation to body weight (BW) and fasting blood glucose (fBG). Fig. 3A showed that db/db mice had higher serom level of resistin than lean control at the age of 5 weeks and 6 weeks. * p<0.05 **p<0.01 *vs* lean control. While serum levels of resistin were comparable between the obese and lean group at the older age. Fig. 3B showed the negative correlation between the circulating resistin level and body weight(BW)(r = −0.515, P = 0.000); Fig. 3C showed the negative correlation between the circulating resistin level and fasting blood glucose (fBG) (r = −0.357, P = 0.002).

At the age of 5 weeks and 6 weeks, db/db mice had higher level of resistin than lean control. Serum levels of resistin were comparable between the db/db obese and lean group at the older age (Fig. 3A). And correlation analysis of all mice showed that serum resistin levels were negatively correlated to the body weight (BW)(r = −0.515, P = 0.000, Fig. 3B) and fasting blood glucose level (r = −0.357, P = 0.002, Fig. 3C). The serum resistin level is not correlated with HOMA-IR (r = −0.194, P = 0.103) or HOMA-B(r = −0.187, P = 0.116) or fasting insulin level(r = −0.035, P = 0.773).

### 2. Time Course of Resistin Production *ex vivo*

**Figure 4:**
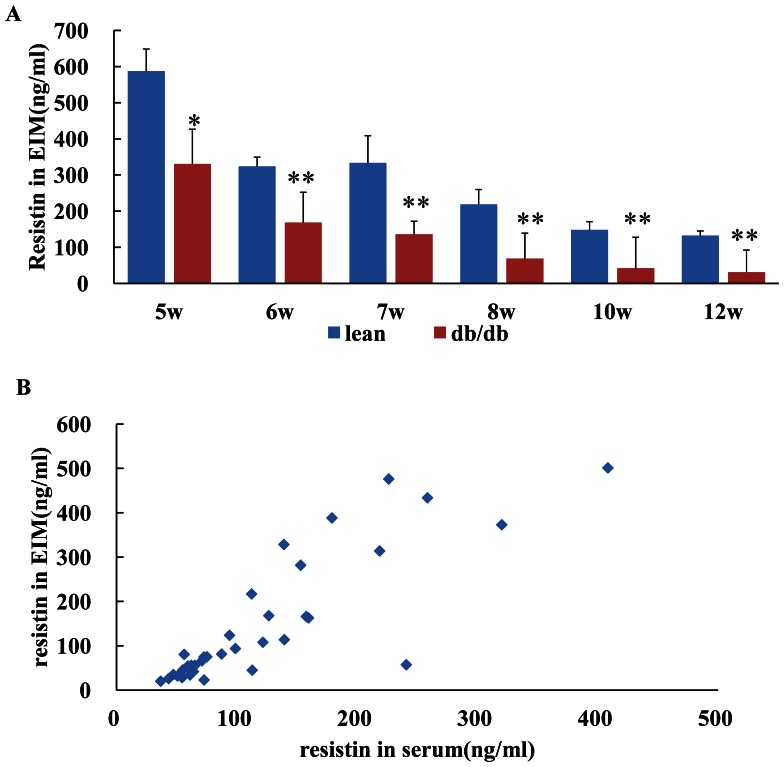
Time course of resistin level in the explants incubation medium (EIM) and its relation to serum resistin level. At each time point, resistin production was decreased in db/db mice(*vs* lean control at the same age *P<0.05, **P<0.01, Fig. 4A); Fig. 4B showed the positive correlation between the resistin level in the explants incubation medium and the resistin level in the serum (r = 0.844 P = 0.000).

At each time point, resistin production from the explants of epididymal WAT was decreased in db/db obese mice compared with lean control mice (Fig. 4A). And in db/db mice, resistin in the explants incubation medium was positively correlated with resistin level in the serum (r = 0.844, P = 0.000, Fig. 4B).

### 3. mRNA Expression of the Resistin Gene in the Epididymal AT

**Figure 5:**
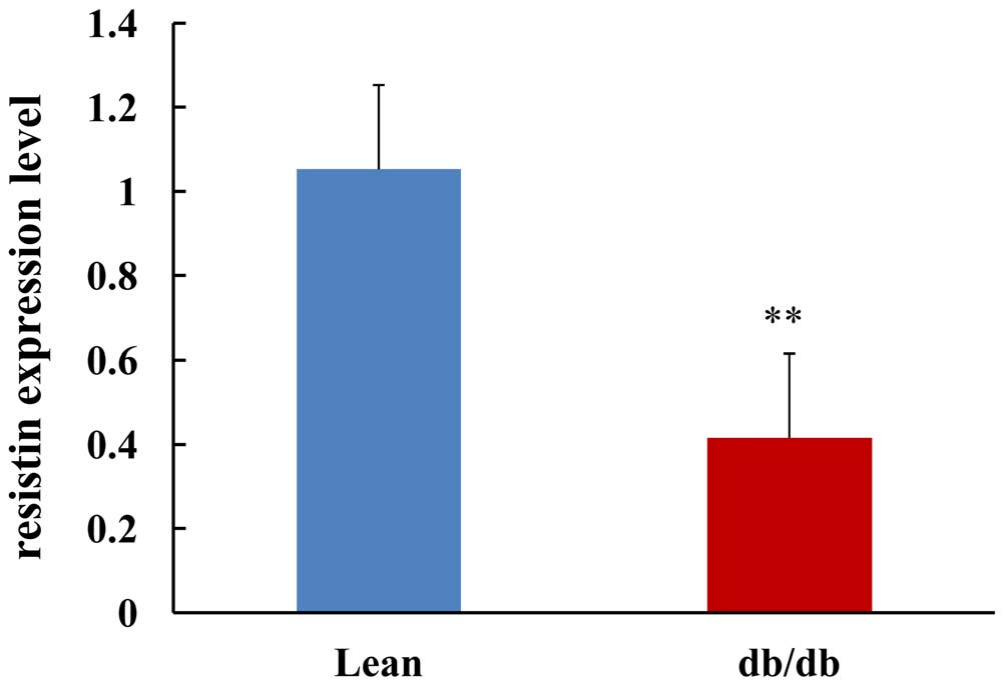
The resistin gene expression in the epididymal adipose tissue at the age of 5 weeks. Total RNA purified from these samples was subjected to quantitative PCR analysis to determine the mRNA levels of resistin. Data were normalized against 18s ribosomal RNA. For comparison, the gene expression levels in the lean control mice were arbitrarily set at 1. **p<0.01 *vs* lean control.

Real time PCR showed that mRNA expression of the resistin gene in the epididymal AT of db/db mice at the age of 5 weeks was decreased by 60.5% compared to their lean controls (P<0.05, Fig. 5).

### 4. The Effect of Rosiglitazone on Resistin Expression and Secretion *in vitro and in vivo*

**Figure 6:**
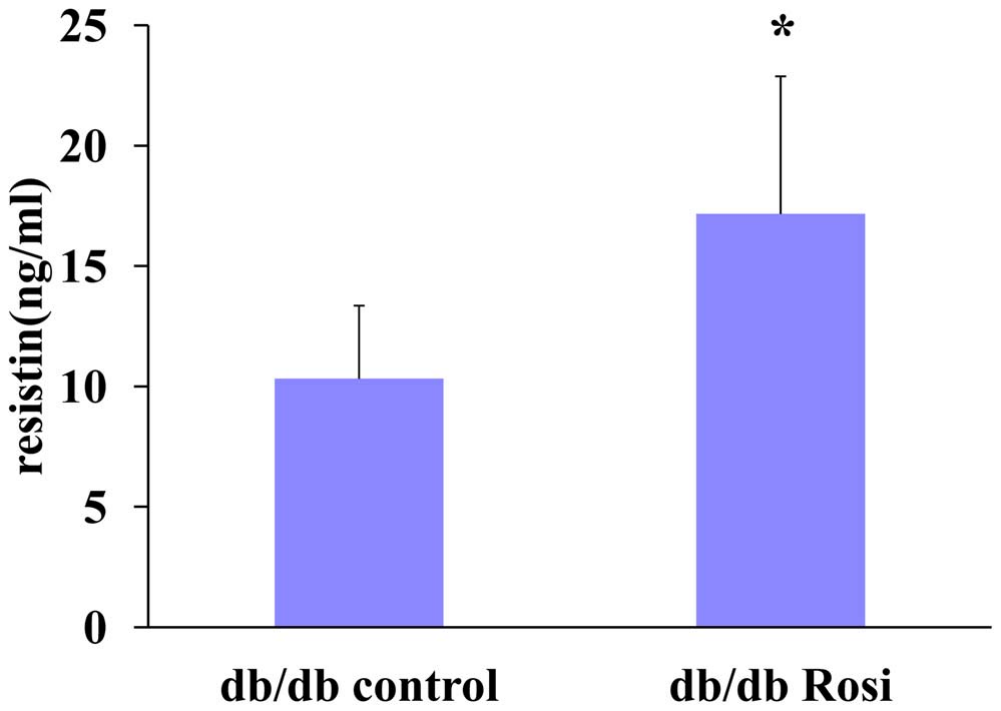
Treatment with Rosiglitazone increased the serum resistin level of db/db mice. Db/db mice were treated with PBS (control) or Rosiglitazone (20 mg/kg/day) by daily intra-gastric gavage for 4 weeks. The db/db mice treated with Rosiglitazone (Rosi) had higher level of serum resistin *vs* control(*P<0.05).

**Figure 7:**
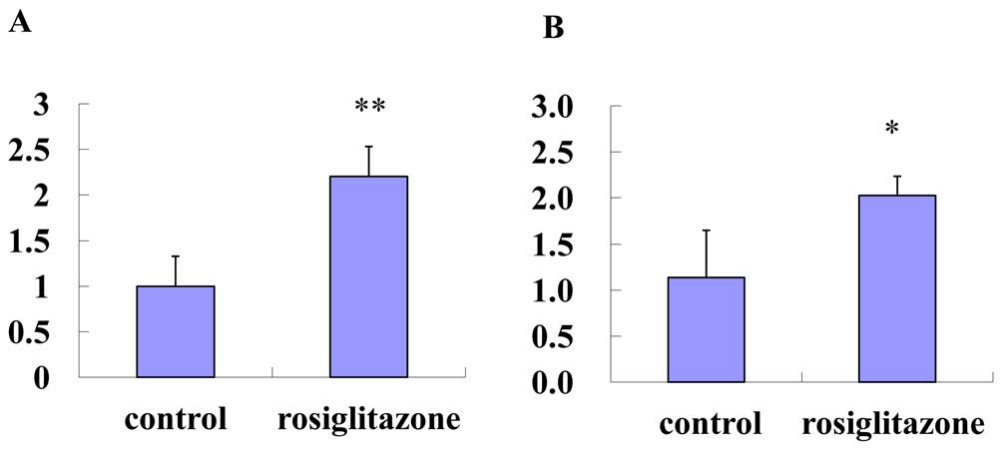
Effects of Rosiglitazone on the protein production and gene expression of resistin in adipocytes differentiated from 3T3-L1. Fully differentiated 3T3-L1 adipocytes were cultured with or without Rosiglitazone (10 uM) for 48 h. The resistin in the conditioned medium was measured (Fig. 7A). Total RNA purified from these samples was subjected to quantitative PCR analysis to determine the mRNA levels of resistin. Data were normalized against 18 s ribosomal RNA. For comparison, the levels of the resistin without Rosiglitazone were arbitrarily set at 1(Fig. 7B). The results were repeated for three times and expressed as mean ±S. *vs* control, *p<0.05**p<0.01.

Notably, treatment of db/db mice with Rosiglitazone for 4 weeks increased the serum resistin levels by 66.4% (P<0.05, Fig. 6) while significantly improved RBG level (7.2±0.95 mmol/L *vs* 21.8±2.2 mmol/L in db/db control, p<0.01). And in adipocytes differentiated from 3T3-L1, the treatment of Rosiglitazone (10 uM) markedly enhanced the secretion of resistin by 120% (P<0.01, Fig. 7A) and its gene expression by 78.1% (P<0.01, Fig. 7B).

### 5. Adiponectin Level in Serum and Production from the Epididymal Adipose Tissue

**Figure 8:**
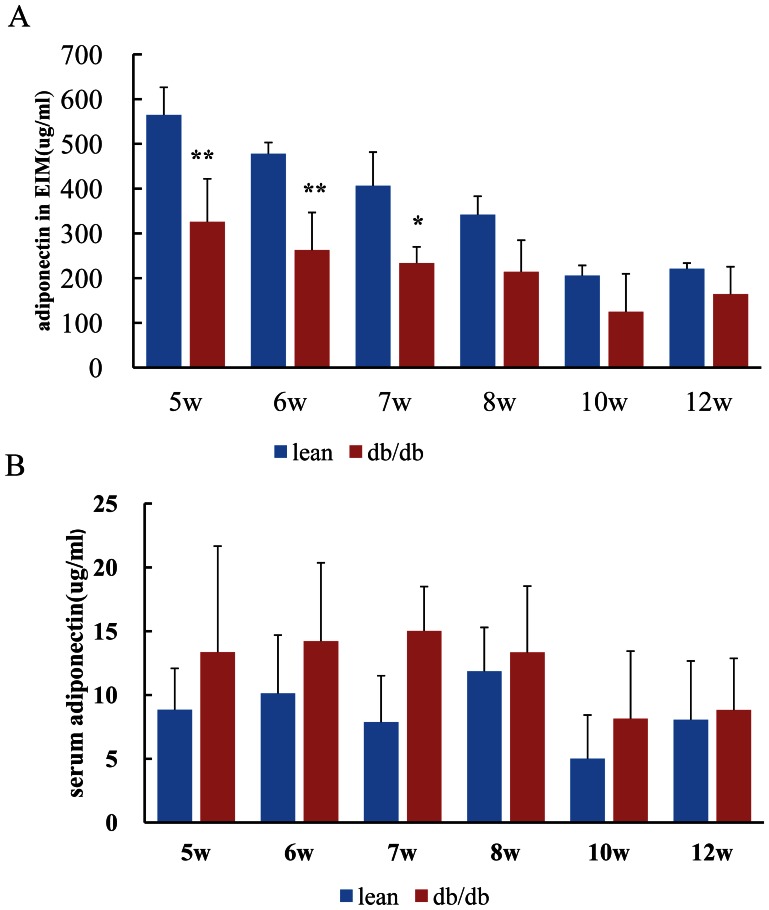
Time course of adiponectin level in the explants incubation medium and serum. At the age of 5w, 6w and 7w, adiponectin level in the explants incubation medium (EIM) of the db/db mice was significantly lower than that of lean mice(P<0.01) (Fig. 8A), indicating that adiponectin production from the epididymal adipose tissue was decreased in db/db mice; Adiponectin in the serum was comparable between db/db mice and lean mice at each point (Fig. 8B).

At each point, adiponectin level in the serum was comparable between db/db mice and lean mice(P>0.05), while adiponectin level in the explants incubation medium (EIM) of the db/db mice was much lower than that of lean mice at the age of 5, 6 and 7 weeks (Fig. 8A,8B).

## Discussion

Obesity is a well-known risk factor for the development of insulin resistance and the metabolic syndrome. Adipose tissue is currently known to secrete a large number of factors with diverse functions. These factors include free fatty acids (FFA) and proteins, termed adipokines, which act in an autocrine, paracrine, or endocrine fashion to control various metabolic functions. Some of these adipocytokines have been implicated in the development of insulin resistance. Accumulating experimental evidence indicates that adiponectin possesses anti-diabetic and anti-inflammatory properties [20], [21]. However, the precise physiological and pathological roles of adipokines, especially resistin, have still not been established unequivocally [1]–[3].

In the present study, we sought to investigate the expression profiles of resistin in db/db mice, a widely used classical model of obesity and diabetes, at different ages (from 5 to 12 weeks) and its association with metabolic parameters. To the best of our knowledge, this study is the first report showing the time course of the serum resistin level and resistin production from the adipose tissue in db/db mice. We found that at the age of 5 weeks and 6 weeks, db/db mice had higher level of resistin than lean control, serum levels of resistin were comparable between the obese and lean group at the older age. But at the age of 5 weeks, the resistin gene expression in the epididymal adipose tissue of db/db mice was decreased by 60.5% compared to their lean controls, and the resistin production from the epididymal adipose tissue was decreased by 43.7% compared to their lean controls. There is a further decrease of resistin production with the older age. At the age of 12 weeks, resistin production in db/db mice was only 23.4% of that from lean control mice. So, the profile of the resistin serum level can be explained by the increased total fat mass along with the decreased expression and production of resistin from the adipose tissue in db/db mice.

The results of the correlation analysis showed that serum resistin levels were negatively correlated to the body weight and fasting glucose level but not correlated with HOMA-IR or HOMA-B or fasting insulin level.

In db/db mice, resistin in the explants incubation medium of the epididymal adipose tissue was positively correlated with resistin level in the serum. This indicated that the epididymal adipose tissue maybe the major source of resistin in db/db mice.

Steppan CM [5] reported that increased circulating resistin levels in diet-induced and genetic obesity could be decreased by rosiglitazon. Way JM et al [11] reported that PPAR-γ agonist such as rosiglitazone increased the resistin expression from WAT in ob/ob mice, but they did not report the change of serum resistin level. In our study, we found that treatment with Rosiglitazone for 4 weeks increased the serum level of resistin in db/db mice. These in vitro results were consistent with the notion that rosiglitazone markedly increased the resistin gene expression and secretion in adipocytes differenciated from 3T3L1.

Therefore, our results suggest that resistin is not an etiological link between obesity and diabetes.

Besides the time courses of serum adiponectin and production from adipose tissue, we performed further experiments to investigate the role of adiponectin in the inflammation of adipose tissue when mice become obese. The results(unpublished data) showed that the reduction in adiponectin production emerges along with insulin resistance and fat mass increase, precedes the macrophage infiltration in the adipose tissue;and epididymal adipose tissue explants incubation medium (EIM )of the db/db mice had stronger chemotaxic effect than their lean controls. Globular adiponectin (20 ug/ml) added to the EIM can weaken their chemotaxic effects on monocytes(THP-1).The decreased adiponectin expression and production may, at least in part, contribute to the chronic inflammation in the white adipose tissue in db/db mice.

In recent years, the numbers of adipokines discovered has expanded rapidly [20]. Adipokines include adiponectin, resistin, visfatin, apelin, retinol binding protein-4, plasminogen activator inhibitor-1, angiotensinogen, vaspin, omentin, and so on. The profiles of adipokines change in response to the change of adipose tissue mass, and are found to play a role in the link between obesity and insulin resistance. But it is still not well known which one maybe the trigger for insulin resistance in obesity.
